# FACE-Rare: Protocol of a randomized controlled trial of a family-centered advance care planning intervention for families of children with rare diseases

**DOI:** 10.1016/j.cct.2026.108430

**Published:** 2026-08-07

**Authors:** Justin A. Yu, Jessica D. Thompkins, Sarah Friebert, Daniel Grossoehme, Justin N. Baker, Heather Gordish-Dressman, Maureen E. Lyon

**Affiliations:** a Division of Pediatric Palliative Medicine and Supportive Care, University of Pittsburgh School of Medicine, Pittsburgh, PA, USA; b Center for Health Outcomes Research Delivery Science, Children’s Research Institute, Children’s National Hospital, Washington D.C., USA; c Haslinger Family Pediatric Palliative Care Division and Rebecca D. Considine Research Institute, Akron Children’s Hospital, Akron, OH, USA; d College of Graduate Studies, Northeast Ohio Medical University, Rootstown, OH, USA; e Division of Quality of Life and Pediatric Palliative Care, Department of Pediatrics, Stanford University and Stanford Medicine Children’s Health, Palo Alto, CA, USA; f Department of Pediatrics, George Washinton University School of Medicine and Health Sciences, Washington D.C., USA

**Keywords:** Pediatric advance care planning, Pediatric palliative care, Pediatric rare disease, Family quality of life, Caregiver appraisal, Intervention, randomized clinical trial

## Abstract

**Background:**

Children with rare diseases and their family caregivers must manage extraordinary care needs, significant symptom burdens, and uncertain prognoses. Pediatric Advanced Care Planning (pACP) is a promising communication-based approach to better support these families. However, empirical evidence regarding the efficacy of pACP among this population is lacking. In response, our team developed and successfully pilot-tested FACE-Rare (FAmily-CEntered Advance Care Planning – Rare Disorders).

**Methods & design:**

This manuscript describes the protocol for a randomized controlled trial evaluating FACE-Rare’s efficacy for improving family quality of life (QOL). We aim to enroll 160 families (child-family caregiver dyads/triads) from three pediatric health systems across the United States. Families will be randomized 1:1 to FACE-Rare or an enhanced treatment as usual control. FACE-Rare is delivered by trained facilitators over three 60-min sessions either virtually (videoconference) or in-person. FACE-Rare utilizes the *Carer’s Alert Thermometer* to identify and address a family’s palliative care needs and the *Respecting Choices: Next Steps pACP* interview to facilitate future medical decision-making. Our primary outcome is family caregiver QOL (caregiver appraisal, emotional distress, and existential well-being). Secondary outcomes include families’ palliative care needs and social connection. An exploratory aim seeks to describe the interactions between sociodemographic characteristics (e.g., household income) and family caregiver QOL.

**Conclusion:**

This is the first randomized controlled trial testing the efficacy of a pACP intervention tailored for families of children with rare diseases. Findings will provide critical evidence about the impact of pACP on family caregiver QOL and medical decision-making for this underserved population.

## 1. Introduction

Children living with rare disease, defined as a health condition affecting fewer than 200,000 persons, are a growing pediatric population that now exceeds 10 million children in the United States [1,2]. Most live with complex chronic health conditions which are complicated by severe disabilities, substantial care needs, high symptom burdens, and uncertain prognoses [3]. As few rare diseases have FDA-approved treatments, children with rare diseases experience high rates of mortality and account for a disproportionately large share of pediatric health care utilization and expenditures [4,5]. As most children with rare diseases live at home, parents and family members (henceforth referred to as family caregivers) provide demanding personal and medical care and complex care coordination, with inadequate support from fragmented health systems [6,7]. These responsibilities and challenges can negatively impact family caregivers’ quality of life (QOL), elevating rates of depression and anxiety, social isolation, loneliness, and financial adversity [8–10]. Given the intrinsic link between family and child well-being, psychosocial interventions designed to improve family caregiver QOL are recognized as an urgent research and clinical priority [11,12].

Pediatric Advanced Care Planning (pACP), a communication approach to prepare families of children with serious illness for future medical decision-making, is a promising strategy to better support family caregivers of children with rare disease [13]. pACP empowers families to consider potential medical care choices and ensure future decisions align with their goals and preferences for their child [14]. Structured pACP interventions have proven efficacious in enhancing this communication process among children with cancer and HIV; improving emotional and spiritual well-being, promoting positive caregiving appraisals, and increasing advanced directive completion [15–17]. However, minimal empirical evidence supporting pACP implementation for families of children with rare diseases exists. Further, children with rare diseases are also often excluded from clinical research due to their clinical heterogeneity and small sample sizes [18]. Consequently, pACP is not standardized nor routinely integrated into the clinical care of children with rare diseases [19].

To address this knowledge and practice gap, investigators developed and refined FACE-Rare (FAmily-CEntered Advance Care Planning – Rare Disorders), an integration of two evidence-based interventions: *Respecting Choices: Next Steps Pediatric ACP*™ and the *Carer’s Alert Thermometer* (CAT)© [20,21]. *Respecting Choices* provides a structured approach to exploring a family’s goals of care for their child, whereas the CAT offers a systematic tool to identify and address a family’s palliative care needs [22–24]. Beta-testing (*n* = 8) and pilot-testing (*n* = 21) of FACE-Rare further refined the intervention based on participant feedback [25,26]. Results demonstrated strong feasibility, acceptability, and improvement in caregivers’ sense of meaning and peace [21], justifying testing FACE-Rare in a fully-powered, intent-to-treat, assessor-blinded, multi-site, randomized controlled trial.

To evaluate the efficacy of FACE-Rare for families of children with rare diseases [27], 160 families (child-family caregiver dyad/triad) will be randomized to the FACE-Rare intervention or enhanced treatment as usual (TAU) comparison condition. Aim 1 is to evaluate FACE-Rare’s efficacy for improving family caregiver QOL at three, six, and twelve months. Aim 2 is to assess FACE-Rare’s efficacy for improving families’ self-reported palliative care needs and social connection across the same time points. Aim 3 is to examine and describe the associations between child sex, family race, and household income with family caregiver QOL, and how these relationships change over time.

## 2. Methods

### 2.1. Trial design

This trial is a prospective, longitudinal, two-armed, single-blinded, multisite randomized controlled trial with an intent-to-treat design. The protocol was developed in accordance with the SPIRIT 2025 (Standard Protocol Items: Recommendations for Interventional Trials) statement (completed checklist in Supplement) [28]. This trial received Institutional Review Board approval from Children’s National Hospital (IRB of record with reliance agreements for all study sites; STUDY00001303), registered with ClinicalTrials.gov (NCT06938542), and will be conducted and reported in accordance with CONSORT guidelines [29]. We will work with the FACE-Rare Community Advisory Board, National Organization for Rare Diseases, and Patient Family Advisory Council to broadly disseminate findings to academic and non-academic audiences (see Supplement).

Child/family caregiver/support person dyads/triads (total *n* = 160 families) will complete self-report assessments at baseline and 3-, 6-, and 12-months post-intervention (Fig. 1). Following baseline assessments, participating families are randomly assigned using a 1:1 allocation ratio to receive either the FACE-Rare intervention or TAU. To ensure group balance, we are using a blocked randomization scheme within four strata. Each of the four study sites (strata) receive 80 randomization slots in which the intervention group’s allocation is based on two randomly assigned block sizes. We are using the Research Electronic Data Capture (REDCap) database to implement this random allocation sequence [30]. Team assessors are blinded to participant group assignment. Participants, intervention facilitators, and participants’ medical teams are unblinded. Participant assessments are administered by intervention facilitators at baseline; and by blinded study team assessors at three-, six-, and twelve-months post-intervention. To ensure participant confidentiality and minimize the collection of protected health information, REDCap will randomly assign generated study identification numbers to all triads (patient, primary caregiver, support person). All physical records are stored in locked, restricted-access facilities, and electronic data is handled in compliance with the Health Insurance Portability and Accountability Act and federal regulations. All study personnel have completed comprehensive training in research ethics and human subjects’ protection. Investigators have safety plan protocols in place (see Supplement) and can provide appropriate referrals if participants endorse thoughts about self-harm.

### 2.2. Participants

Investigators are enrolling families of children with rare diseases unable to participate in end-of-life care decisions themselves. Operationally, “family” is defined as a child, their legal guardian(s), and a support person chosen by the legal guardian (e.g., second parent). *Child inclusion criteria* includes: (1) age ≥ 1 to ≤18 years at enrollment, (2) inability to participate in end-of-life decision-making due to age, communication disorder, and/or intellectual disability, and (3) have a rare disease (any disease included in the Genetic and Rare Disease Information Center Database) associated with multiple morbidities [31]. *Child exclusion criteria* includes: (1) infants (age < 1 year), (2) in foster care, (3) admitted to intensive care unit, and (4) have an active “Do Not Resuscitate” or “Allow Natural Death” order at the time of enrollment. *Family caregiver inclusion criteria* include: (1) ≥18 years of age at enrollment; (2) child’s legal guardian; (3) not known to have intellectual disability; and (4) not actively homicidal, suicidal, or psychotic at time of enrollment. Investigators are excluding families who participated in the development, beta-testing, and pilot trial of the FACE-Rare intervention. Investigators are including participants with limited English proficiency. Additionally, families of children with a primary diagnosis of autism, cystic fibrosis, cancer, HIV, Duchenne’s Muscular Dystrophy, and Sickle Cell Disease are excluded to prioritize including families of children historically excluded from palliative care research due to condition-specific requirements. Furthermore, children with conditions like cystic fibrosis more commonly have access to well-established, disease-specific medical home infrastructures which provide comprehensive multidisciplinary care; including these sub-populations could limit evaluating the FACE-Rare intervention among rare disease populations lacking access to comprehensive care models.

### 2.3. Recruitment procedures

Participants are being recruited across three tertiary-care academic pediatric health centers: (1) Children’s National Hospital (coordinating center), (2) Akron Children’s Hospital, and (3) UPMC Children’s Hospital of Pittsburgh. Within each site, recruitment will occur from subspecialty clinics and service teams that care for children with medical complexity and/or serious illness such as pediatric complex care, palliative care, neurodevelopmental disorders, and medical genetics. Study team members identify potentially eligible families through review of electronic medical records and assistance from local clinical teams. To minimize gatekeeper bias, site PIs conducted educational forums to discuss study details and address legitimate reasons for clinician-directed refusals. For potentially eligible families, a member of the child’s medical team uses a recruitment flyer to obtain verbal permission for a study team member to approach the potential participant. Upon obtaining permission, a team member will approach a potential participant in-person, telephone, or schedule a video teleconference to provide full study details using a standardized introductory script. For interested families, study team members then conduct eligibility verification and obtain informed consent electronically via REDCap [30]. Families have the option of completing eligibility screening and informed consent at the time of approach or later. Participants will receive $25 for completing each study session and assessment ($175 total intervention arm; $100 total TAU).

### 2.4. Intervention & TAU

Participants continue to receive standard care from their medical team. At baseline, participants in both study arms complete the Biomedical Research Informatics Computing System (BRICS) Social Determinants of Health questionnaire developed by the National Institute on Nursing Research (NINR) [32,33]. If unmet basic needs are identified, study team members refer families to appropriate resources and services at their study site [34]. Both arms also receive a booklet created by the NINR entitled, “Palliative Care for Children: Support for the Whole Family When Your Child Is Living with a Serious Illness.” [35].

#### Intervention: Family Centered Advance Care Planning –

FACE-Rare is an intervention for family surrogate decision-makers of children with rare disease. FACE-Rare is built on transactional stress and coping theory, with an emphasis on promoting active problem solving, and integrates caregiver illness representation theory, which posits that one’s beliefs about illness have five dimensions: identity, cause, timeline, consequences, and cure/control [37–39]. In other words, family caregivers’ perceptions about their child’s illness influence their use of various coping strategies, which then affect their appraisal of the caregiving role [40,41].

FACE-Rare integrates two evidence-based interventions into three sessions with a trained facilitator (Fig. 1). FACE-Rare Sessions 1 and 2 focus on creating and implementing a shared action plan to identify and address a family’s palliative care needs. In Session 1, study facilitators use the *Carer Alert Thermometer* (CAT) to identify and prioritize a family’s needs across multiple palliative care domains [24]. Families and facilitators then collaboratively create a shared action plan to address their highest-priority needs. In Session 2 (one to two weeks after Session 1), facilitators review what was accomplished from the shared action plan and facilitate additional problem-solving to address obstacles encountered. The CAT tool reflects a collaboration to adapt the *Pediatric Carer Support Needs Assessment Tool* [42,43].

In Session 3, participants meet with the facilitator to complete a video-recorded *Respecting Choices: Next Steps pACP* interview (Table 1). In this conversation, interventionists facilitate discussion about a child’s medical conditions and family members’ fears, values and beliefs, and hopes for the future [44]. Summarizing this information, interventionists facilitate a conversation to consider potential medical scenarios to prepare families for future medical decisions. Families are asked to choose one of three medical treatment approaches should their child experience a critical health event requiring a prolonged hospital stay, ongoing medical interventions, and prognostic uncertainty: (1) life prolongation by all medically effective means; (2) continuation of treatments with specific limitations; or (3) cessation of all medical treatments except those aiming to maximize child’s comfort and permit a natural death. Upon completion of the pACP interview, and with a family’s permission, the study team uploads the pACP document to the child’s electronic health record and shares it with the child’s medical team (facilitator emails brief summary and pACP document to family-chosen clinicians).

All three sessions of the FACE-Rare intervention are designed for either telehealth (via HIPPA-compliant videoconference software) or in-person delivery by trained facilitators. Sessions will be conducted individually at a target rate of one session per week. Those without home access to the internet and/or electronic devices capable of videoconferencing will be loaned electronic tablets with remote internet service.

#### Enhanced Treatment as Usual (TAU) –

Participants randomized to TAU will continue to receive their child’s standard medical care and a the NINR booklet, “Palliative Care for Children: Support for the Whole Family When Your Child Is Living with a Serious Illness.” This brochure explains how pediatric palliative care can assist children with serious illness, and their families, by addressing physical symptoms and offering emotional, social, and spiritual support at any stage of a child’s clinical trajectory [35]. Investigators chose enhanced TAU as the comparison condition to minimize burden on participating families. This reflected community and clinician feedback that an active comparator would be too burdensome. Additionally, current standard practice for children with life-limiting illnesses is to defer initiation of pACP discussions until a medical crisis [44,45].

### 2.5. Facilitator training & fidelity and safety monitoring

A three-day in-person training course for all team members was conducted at Children’s National Hospital prior to opening enrollment. During this training, the trial manual of operating procedures, protocols, and procedures were reviewed. Study facilitators underwent CAT training through didactic activities, discussion, and role play opportunities. Study facilitators also completed the Respecting Choices^®^ Next Steps ACP Facilitator Certification Course which consisted of pre-training online modules and in-person didactics and simulation activities with standardized patients (>6 h). Facilitators were required to obtain this certification prior to interviewing family participants.

To ensure intervention fidelity, adherence to trial protocols and regulatory procedures, and to address challenges as they arise, the study Principal Investigator (PI) and coordinating study staff conduct monthly meetings with the entire study team members and annual site visits (or more often if needed). Additionally, the study PI will review all video recordings of Session 3 within one week of the study visit for adherence and competence. Site investigators meet with the site trial team weekly. Lastly, we plan to conduct a remote booster session (study year 3–4) with the entire study team to review study protocols and procedures.

An external safety monitoring committee consisting of two clinicians with pediatric palliative care expertise, a social worker, and clinical psychologist has been established. This committee, chaired by a senior nurse researcher, will meet with the study PI annually, or more often if needed, to review study progress, safety information, and any reports of adverse events (AE) or serious adverse events (SAE). (see Supplement).

### 2.6. Data collection & retention strategies, measures, and medical & demographic variables

Participants complete outcome measures one-on-one (in-person or virtually) with study facilitators (baseline) and blinded study team assessors at 3-, 6-, and 12-months post-intervention. All responses are stored in REDCap. To minimize survey fatigue, optimize participant understanding, and support longitudinal retention, study team members read aloud all survey items [47]. Study team assessors have a 1-month window for completing 3-, 6-, and 12-month post-intervention assessments. The full assessment battery was refined in collaboration with a family caregiver advisory panel using community-based participatory research principles. Pilot testing demonstrated mean completion time of 27 min.

All outcome measures (Table 2) were selected for their reliability, validity, and presence of normative data. Primary outcome measures assess family caregiver QOL: Family Appraisal of Caregiving for Palliative Care [46], Generalized Anxiety Disorder-7 (GAD-7) [47], Patient Health Questionaire-9 (PHQ9) [48], and Functional Assessment of Chronic Illness Therapy – Spiritual Well-Being Scale (FACIT-Sp-12) [49]. Secondary outcomes and measures include: Carer Alert Thermometer (CAT) [24], BRICS NINR Social Determinants of Health (BRICS SDOH) [33], Brief-Multidimensional Measure of Spirituality and Religion (BMMRS) [50], family home residence rurality (determined by HRSA: Rural Health Grants Eligibility Analyzer) [51], advanced care planning documentation, medical decision-making stress, and participant satisfaction.

Investigators are collecting information regarding child health care utilization (e.g., number of hospital admissions, time between palliative care referral and death) using standard data abstraction procedures. Investigators are also collecting the following child and family demographic covariates (Table 2).

## 3. Aims & Hypotheses

### AIM 1.

To determine the efficacy of FACE-Rare on family caregiver QOL (caregiver appraisals, emotional distress, spiritual well-being) at 3-, 6- and 12-month follow-up. Controlling for child and family characteristics (e.g., child age, gender and race of family and child) investigators hypothesize that FACE-Rare, compared to TAU, will: increase positive appraisals of caregiving, decrease caregiver strain, decrease caregiver emotional distress (symptoms of anxiety and depression), and increase caregiver feelings of peace.

### Aim 2:

To assess the effect of FACE-Rare on families’ palliative care needs at 3-, 6-, and 12-month follow-up and changes in palliative care needs across the study period. Investigators hypothesize that compared to participants assigned to TAU, FACE-Rare participants will report lower endorsed palliative care needs, lower intensity of palliative care needs over time, and increased social connection, regardless of household income or rural vs. urban setting.

### AIM 3.

To determine if the intersections of child-sex, family-race, and household income are associated with family caregiver QOL over time. Investigators will examine the interaction between family race and household income, hypothesizing that income-related disparities in family QOL over time will differ significantly by family race. Investigators will also explore the interaction between child-sex and household income, hypothesizing that income-based disparities in family QOL over time will differ significantly by child sex.

### 3.1. Statistical analysis

Descriptive statistics will characterize the study sample at baseline. Continuous variables will be summarized using medians and interquartile ranges, and categorical variables using frequencies and percentages. Baseline comparability between the FACE-Rare and TAU groups will be assessed using Chi-square tests for categorical variables and Mann-Whitney *U* tests for continuous variables. Analyses focusing on the effect of FACE-Rare compared to TAU will follow the intent-to-treat principle - all participants will be analyzed as part of the group to which they were assigned, regardless of protocol adherence or attrition. Any additional analyses will be determined as necessary only following the completion of 12-month post-intervention data collection.

#### Sample Size:

Power/sample size calculations were computed using a simulation scheme [58,59]. Simulation parameters were informed by data collected from our FACE-Rare pilot study, end-of-life care data from prior FACE pACP studies, attrition estimates, and estimated suicidal or homicidal ideation and/or psychosis [21]. Sixty-six triads are required to detect a statistically significant FACE-Rare effect at alpha level of 0.05, across all time observations, for Aim 1 and 2 outcomes with 80% power. Investigators also calculated that 135 triads were necessary for the same statistical power and alpha level to detect treatment effects at specific study timepoints. For Aim 3, 160 triads provide statistical power of 0.71 at the 0.05 alpha level to detect any significant interactions between child-sex, family-race, and household income on family QOL. Therefore, investigators plan to enroll and randomize a total sample size of 160 triads.

#### Aim 1:

To determine the efficacy of FACE-Rare on family QOL at 3-, 6-, and 12-month follow-up, investigators will utilize Generalized Linear Mixed-Effects Models (GLMM) specifying a Gamma distribution with a log link function in anticipation of the skewed nature of QOL data [60,61]. Given the hierarchical nature of our data, GLMMs will incorporate a nested random effects structure with random intercepts to account for clustering within families and repeated measures within the same parent over time [62]. Fixed effects will include treatment group, timepoint, and time- treatment group interaction. Based on our pilot trial results, investigators will include child age, child and parent sex, parent race, and baseline religious coping in all final GLMMs [21]. To ensure model parsimony, additional time-invariant covariates will be selected using L1 penalized variable selection [63]. Model’s goodness of fit will be evaluated using marginal and conditional random effects residuals and 95% bootstrap confidence intervals will be calculated with a minimum of 1000 iterations. [64,65] If significant main or interaction effects are detected, investigators will perform planned contrasts on estimated marginal means to compare treatment effects at the specific follow-up time points. The primary test of FACE-Rare’s efficacy will be the fixed-effect time-by-treatment interaction - assessing whether the longitudinal trajectory of family QOL differs significantly between treatment groups. This approach for GLMM construction will be repeated independently for each measure of family QOL. To evaluate potential heterogeneity of treatment effect by disease severity, pre-specified secondary analyses will conduct identical GLMMs stratified by a child’s (1) neurodegenerative disease status, (2) baseline medical technology utilization, (3) baseline subspecialty palliative care involvement, and (4) number of hospital days in the prior 30 days. Stratified estimated marginal means and treatment effect trajectories will compare intervention efficacy across these markers of clinical severity.

#### Aim 2:

Aim 2 analyses will utilize GLMMs constructed using modelling approaches described in Aim 1 to evaluate changes in secondary outcomes, palliative care needs (CAT) and social connection (BRICS SDOH), across study timepoints by treatment group. Our secondary outcomes involve ordinal data and data distributions from our prior pilot trial suggests that responses should be dichotomized. Investigators will consider either jointly modelling all CAT-based outcomes assuming multinomial distribution or independently modelling each CAT-based outcome using logistic mixed-effects regression [66,67]. Alternatively, investigators will utilize cumulative link GLMMs if CAT-based data are analyzed as ordinal outcomes [68]. For social connection, investigators will similarly consider between dichotomization and ordinal structure of BRICS SDOH data based on data distribution. These GLMMs will also control for participants’ household income and rurality (HRSA Rural Health Grants Eligibility Analyzer).

#### Aim 3:

To evaluate the intersection of family-race and household income with family QOL over time, investigators will employ the GLMM approach as described in Aim 1, however models will be extended to include an interaction term between family race and household income (2023 Federal Poverty Level). To examine the intersection of child-sex and household income with child health care utilization (e.g., hospitalizations over 12-month study period), investigators will include an interaction term between child-sex and household income in similarly constructed GLMMs.

#### Missing Data:

If data are “missing at random,” investigators will address missing covariate data using multiple imputation by fully conditional specifications [69]. If the “missing at random” assumption is untenable, investigators will identify reasons for missingness, and consider adjusting the GLMMs’ covariance structure to address the specific missingness mechanism [70].

## 4. Discussion

This is the first trial to systematically test the efficacy of FACE-Rare, a pediatric palliative care intervention specifically designed to assess the needs of families of children with rare diseases and test a pACP intervention. FACE-Rare moves beyond traditional end-of-life planning by providing a holistic approach to ACP which addresses families’ immediate care needs and supports future medical decision-making for their child – a new ACP paradigm referred to as the “caring umbrella.” [71] Findings from this trial will produce critical insights into whether a family-centered communication intervention can improve family caregiver QOL outcomes compared to treatment as usual: (1) positive appraisal of caregiving; (2) caregiver strain; (3) caregiver emotional distress (symptoms of anxiety and depression); and (4) feelings of peace and meaning. Our findings will also inform future effectiveness and implementation research, with the long-term objective of strengthening pediatric clinicians’ capacity to provide high-quality, family-centered care to families of children facing profound prognostic uncertainty, intensive care demands, and persistent gaps in health system support. Findings may support the importance of clear and timely communication between clinicians and families of medically fragile children, for whom clinical decision-making is often complex, emotionally charged, and evolving over time.

FACE-Rare is an innovative approach as it shifts pACP towards a proactive, values-based strategy that empowers clinicians to support families navigating the complexities of rare medical conditions [72,73]. By incorporating the CAT, FACE-Rare provides a holistic and scalable process to identify and address a family’s palliative care needs and social drivers of health [24,42]. FACE-Rare uses telemedicine to overcome traditional barriers to access related to geography and transportation.

Beyond its clinical potential, FACE-Rare is also well-positioned to advance the pediatric palliative care literature. By enrolling a group traditionally disenfranchised from pediatric research, this trial answers national calls to increase clinical research inclusivity and provides much-needed data regarding family caregivers’ QOL and psychosocial outcomes [4,42]. This trial will also provide essential insights into this unique population’s palliative care needs and clarify how key social drivers of health influence family caregiver QOL. As advancements in medical treatments and technologies improve survival among children with rare diseases, they also create a distinct caregiver population facing high-intensity demands and challenges over protracted timelines [10,13,74]. Findings will inform the development of sustainable palliative care resources and infrastructure to support families over their child’s lifetime.

Limitations include a relatively small, heterogeneous sample of diagnoses, which may limit our ability to detect smaller effect sizes or generalize findings to specific diseases [75]. Unblinded participants introduces the potential for “resentful demoralization,” [76] a form of bias induced when participants are likely to have strong treatment arm preferences and do not receive their preferred treatment. The medical fragility of this pediatric population may result in attrition or interruptions in study participation, requiring flexible protocol adherence that balances rigorous data collection with family support [77].

In summary, this trial of FACE-Rare aims to improve caregiver QOL and address the needs of families of children with rare diseases by demonstrating the efficacy of a targeted and scalable model of pACP. FACE-Rare represents a significant step forward in the science of pACP and centers the perspectives and needs of an often-overlooked patient and family caregiver population.

### Studies in human

This study was performed in compliance with relevant laws, regulatory frameworks and guidelines where the research took place. This study was approved by the Children’s National Hospital (Approval No. STUDY00001303).

## Supplementary Material

Supplementary Material

## Figures and Tables

**Fig. 1.**
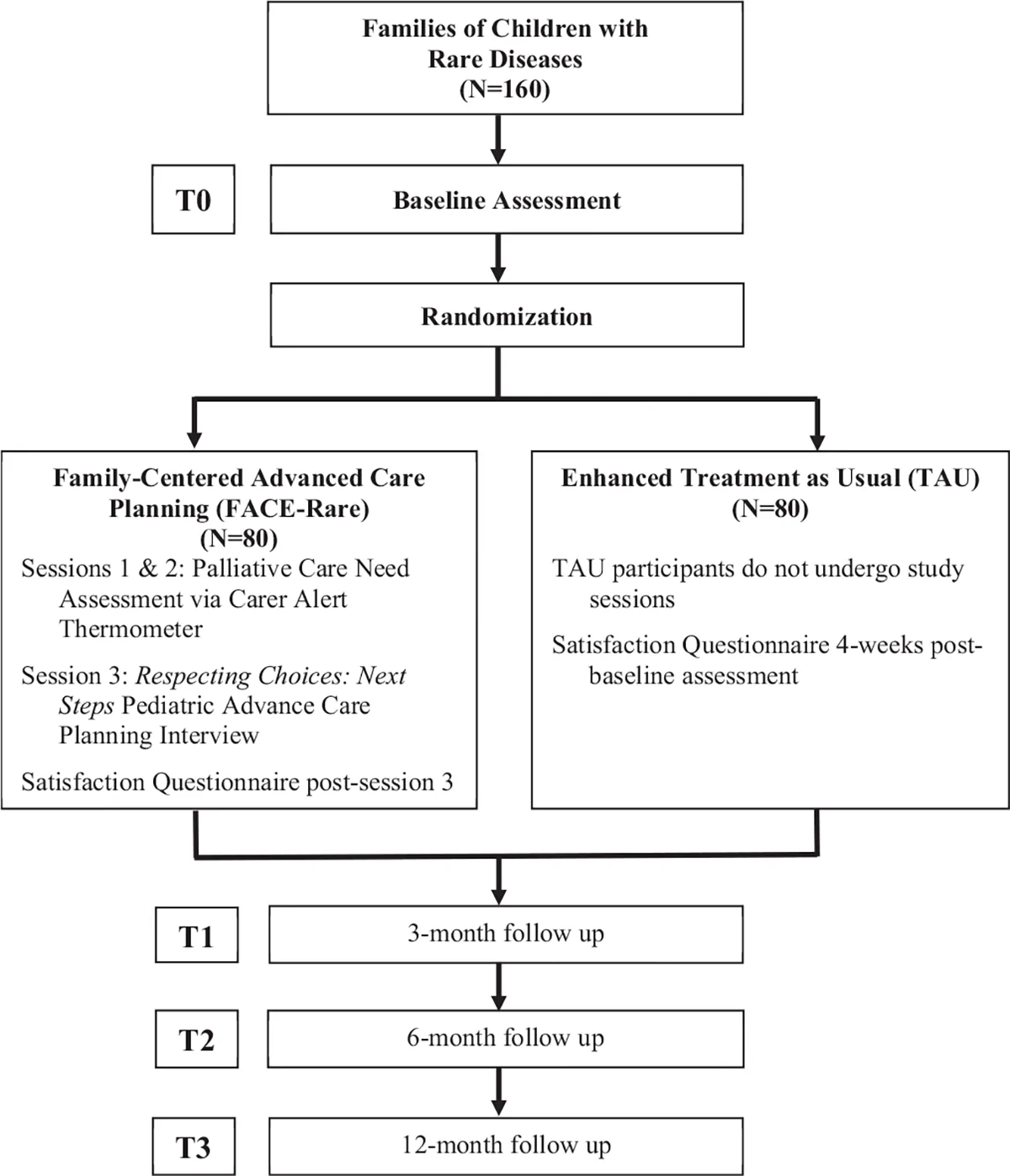
Study Flow Diagram.

**Table 1:** FACE-Rare Session 3 - *Next Steps: Respecting Choices pACP*^®^.

Foundation	pACP for children with serious illness unable to participate in healthcare decision-making
Goals	To facilitate conversations with family caregivers about their child’s medical condition, fears, values and beliefs (including religious/spiritual) and hopes for the future. To prepare the family for future medical decisions if their child experiences an acute critical illness.
Process	**Stage (S) 1** assess family’s understanding of child’s illness: hopes, fears, concept of living well; **S2** explore family’s experiences with child’s hospitalization; **S3** review goals of care and set the stage for future healthcare decisions. **S4** Explore goals for future healthcare, using a situation-based Advance Care Plan document; **S5** Questions for child’s provider identified and written on post-card; **S6**: Follow-up Plan.

**Table 2:** 

Ooutcome Measures	Time	Description

Primary Outcomes: Family Caregiver QOL		
Family Appraisal of Caregiving for Palliative Care [46]	BL, 3, 6, 12 mo	**Caregiver appraisal**: caregiver strain, positive caregiving appraisals, caregiver distress, family well-being in past two weeks. 25 items. Subscales will be used in the analysis.
Generalized Anxiety Disorder-7 (GAD-7) [47]	BL, 3, 6, 12 mo	**QOL:** emotional health-anxiety symptoms. 7 items. Total score will be used in the analysis.
Patient Health Questionaire-9 (PHQ9) [48,52]	BL, 3, 6, 12 mo	**QOL:** emotional health-depressive symptoms. 9 items. Total score will be used in the analysis. A yes response to question 9 (self-harm) will trigger a referral.
FACIT-Spirituality-12 [48]	BL, 3, 6, 12 mo	**QOL**: spiritual (subscales: meaning/purpose, peace). Is culturally sensitive to those with non-theistic beliefs. 12-items. Peace subscale will be used in analysis.
Secondary Outcomes		
Carer Alert Thermometer (CAT) [23,53]	BL, 3, 6, 12 mo	**Palliative Care Needs:** The CAT is an evidence-based, comprehensive tool comprising 12 questions (broad areas of support need), used to identify two domains of unmet support needs, the current caring situation and the health and well-being of the caregiver. There is a traffic light scoring system so caregivers can rate their support need as green (low), amber (medium), and red (high) indicating level of need and potential risk each alert poses to the caregiving situation.
BRICS NINR Social Determinants of Health (BRICS SDOH) [33]	BL, 3, 6, 12 mo	SDOH are the conditions in which people are born, grow, live, work and age….” 32 items. **Social connection score** (how often, talk on telephone with family, friends, neighbors; get together; attend church or religious services; belong to clubs or organizations; marital partner status) and **2023 Federal Poverty Level for household income**. Health insurance and housing insecurity will be used in secondary analysis.
Brief Multidimensional Measure of Spirituality and Religion (BMMRS) [50]	BL, 3, 6, 12 mo	**Religious Coping**: 5 items from our previous research: attend religious services, feel God’s presence, pray privately, identify as religious, identify as spiritutal [12,58,59].
HRSA: Rural Health Grants Eligibility Analyzer [51]	BL, 3, 6, 12 mo	**Urban vs. rural setting.** Address of participant is entered into the online analyzer, and it determines if this is a rural or urban setting which will be recorded in the data base.
Advance Care Documentation for Children with Rare Diseases	BL, 12 mo	**ACP Documentation in EHR & Decisional preferences**- to continue all treatments; to continue all treatments with exceptions noted; to stop all treatments but comfort care.
Standardized Data Abstraction Operationally defined [54,55]	BL, 3, 6, 12 mo or close-out	**Child healthcare utilization**: initiation of palliative care consultations, # of days in palliative care prior to death, hospitalizations, Emergency Department visits, intensive care unit use, surgeries.
Role stress [56]	BL, 3, 6, 12 mo	Visual analogue scale 0–100. “How stressful is it for you to make medical decisions for your child?” 1 item.
Time-Invariant Covariates		
Demographic Questionnaire	BL	Age, sex/gender, race, ethnicity of family caregiver and child, marital status, education, number of persons in household, household income.
Process Measures		
Satisfaction Questionnaire [57]	Timing: Intervention arm (immediately post-session 3); TAU arm (3–4 weeks post-baseline assessment). Study specific process measure to assess adverse events and benefit/burden of participation. 12 items.	

**Abbreviations:** QOL, Quality of Life; BL, baseline; mo, month; NINR, National Institute of Nursing Research; SDOH, social determinants of health; TAU, treatment as usual.

## Data Availability

Data will be made available on request.

## Appendix A. Supplementary data

Supplementary data to this article can be found online at https://doi.org/10.1016/j.cct.2026.108430.

## Abbreviations:

QOL: Quality of Life
pACP: Pediatric Advanced Care Planning
FACE-Rare: Family-Centered Advance Care Planning – Rare Disorders
CAT: Carer’s Alert Thermometer (CAT)©
TAU: Enhanced Treatment as Usual
SPIRIT: Standard Protocol Items: Recommendations for Interventional Trials
REDCap: Research Electronic Data Capture
BRICS: Biomedical Research Informatics Computing System
NINR: National Institute on Nursing Research
AE: Adverse Event
SAE: Serious Adverse Event
GAD-7: Generalized Anxiety Disorder-7
PHQ-9: Patient Health Questionaire-9
FACIT-Sp-12: Functional Assessment of Chronic Illness Therapy – Spiritual Well-Being Scale
BRICS NINR SDOH: BRICS NINR Social Determinants of Health
HRSA: Health Resources and Services Administration
BMMRS: Brief Multidimensional Measure of Spirituality and Religion

## References

[1] Rare Diseases FAQ, National Institutes of Health, 2024.

[2] Dumbuya JS, Zeng C, Deng L, , The impact of rare diseases on the quality of life in paediatric patients: current status, Front. Public Health 13 (2025) 1531583. 10.3389/fpubh.2025.1531583 PMC11973084 40196857

[3] Tisdale A, Cutillo CM, Nathan R, , The IDeaS initiative: pilot study to assess the impact of rare diseases on patients and healthcare systems, Orphanet J. Rare Dis. 16 (1) (2021) 429. 10.1186/s13023-021-02061-3 PMC8532301 34674728

[4] Adams LS, Miller JL, Grady PA, The Spectrum of caregiving in palliative Care for Serious, advanced, Rare Diseases: key issues and research directions, J. Palliat. Med. 19 (7) (2016) 698–705. 10.1089/jpm.2015.0464 PMC4931351 27249541

[5] The Lancet Global H, The landscape for rare diseases in 2024, Lancet Glob. Health 12 (3) (2024) e341. 10.1016/S2214-109X(24)00056-1 38365397

[6] Bally JMG, Smith NR, Holtslander L, , A Metasynthesis: uncovering what is known about the experiences of families with children who have life-limiting and life-threatening illnesses, J. Pediatr. Nurs. 38 (2018) 88–98. 10.1016/j.pedn.2017.11.004 29357986

[7] Teicher J, Moore C, Esser K, , The experience of parental caregiving for children with medical complexity, Clin. Pediatr. (Phila) 62 (6) (2023) 633–644. 10.1177/00099228221142102 PMC10676025 36475307

[8] Lai X, Jiang Y, Sun Y, Zhang Z, Wang S, Prevalence of depression and anxiety, and their relationship to social support among patients and family caregivers of rare bone diseases, Orphanet J. Rare Dis. 18 (1) (2023) 18. 10.1186/s13023-022-02611-3 PMC9878818 36703212

[9] Landfeldt E, Lindgren P, Bell CF, , Quantifying the burden of caregiving in Duchenne muscular dystrophy, J. Neurol. 263 (5) (2016) 906–915. 10.1007/s00415-016-8080-9 PMC4859858 26964543

[10] Pelentsov LJ, Laws TA, Esterman AJ, The supportive care needs of parents caring for a child with a rare disease: a scoping review, Disabil. Health J. 8 (4) (2015) 475–491. 10.1016/j.dhjo.2015.03.009 25959710

[11] Coleman CL, Morrison M, Perkins SK, Brosco JP, Schor EL, Quality of life and well-being for children and youth with special health care needs and their families: a vision for the future, Pediatrics 149 (Supplement 7) (2022) e2021056150G. 10.1542/peds.2021-056150G 35642872

[12] Administration for Community Living, National Strategy to Support Family Caregivers, U.S. Department of Health and Human Services, 2022.

[13] Dellve L, Samuelsson L, Tallborn A, Fasth A, Hallberg LR, Stress and well-being among parents of children with rare diseases: a prospective intervention study, J. Adv. Nurs. 53 (4) (2006) 392–402. 10.1111/j.1365-2648.2006.03736.x 16448482

[14] Institute of Medicine. Committee on Approaching Death: Addressing Key End of Life Issues, Dying in America: Improving Quality and Honoring Individual Preferences near the End of Life, The National Academies Press, Washington, DC, 2015. 25927121

[15] Lusney N, van Breemen C, Lim E, Pawliuk C, Hussein Z, Pediatric advance care planning: a scoping review, Children (Basel) 10 (7) (2023). 10.3390/children10071179 PMC10378635 37508676

[16] Lyon ME, Jacobs S, Briggs L, Cheng YI, Wang J, A longitudinal, randomized, controlled trial of advance care planning for teens with cancer: anxiety, depression, quality of life, advance directives, spirituality, J. Adolesc. Health 54 (6) (2014) 710–717. 10.1016/j.jadohealth.2013.10.206 24411819

[17] Lyon ME, Garvie PA, Briggs L, , Is it safe? Talking to teens with HIV/AIDS about death and dying: a 3-month evaluation of family centered advance care (FACE) planning - anxiety, depression, quality of life, HIV AIDS (Auckl). 2 (2010) 27–37. 10.2147/hiv.s7507 PMC3218704 22096382

[18] Cohen E, Kuo DZ, Agrawal R, , Children with medical complexity: an emerging population for clinical and research initiatives, Pediatrics 127 (3) (2011) 529–538. 10.1542/peds.2010-0910 PMC3387912 21339266

[19] Lotz JD, Jox RJ, Borasio GD, Führer M, Pediatric advance care planning: a systematic review, Pediatrics 131 (3) (2013) e873–e880. 10.1542/peds.2012-2394 23400610

[20] Lyon ME, Thompkins JD, Fratantoni K, , Family caregivers of children and adolescents with rare diseases: a novel palliative care intervention, BMJ Support. Palliat. Care 12 (e5) (2022) e705–e714. 10.1136/bmjspcare-2019-001766 PMC8040699 31345846

[21] Lyon ME, Fraser JL, Thompkins JD, , Advance care planning for children with Rare Diseases: a pilot RCT, Pediatrics 153 (6) (2024). 10.1542/peds.2023-064557 PMC11153326 38699801

[22] Hammes BJ, Klevan J, Kempf M, Williams MS, Pediatric advance care planning, J. Palliat. Med. 8 (4) (2005) 766–773. 10.1089/jpm.2005.8.766 16128650

[23] Knighting K, O’Brien MR, Roe B, , Development of the carers’ alert thermometer (CAT) to identify family carers struggling with caring for someone dying at home: a mixed method consensus study, BMC Palliat. Care 14 (2015) 22. 10.1186/s12904-015-0010-6 PMC4427956 25935715

[24] Aoun SM, Stegmann R, Deleuil R, , “It is a whole different life from the life I used to live”: assessing parents’ support needs in paediatric palliative care, Children (Basel) 9 (3) (2022). 10.3390/children9030322 PMC8947323 35327694

[25] Younge T, Moore H, Thompkins JD, Lyon ME, Parental goals of care for children with rare diseases: a content analysis of pediatric advance care planning conversations, Am. J. Hosp. Palliat. Care 43 (8) (2025) 851–860. 10499091 406013017. 10.1177/10499091251356237 PMC12710983

[26] Fratantoni K, Livingston J, Schellinger SE, Aoun SM, Lyon ME, Family-Centered advance care planning: what matters Most for parents of children with Rare Diseases, Children (Basel) 9 (3) (2022). 10.3390/children9030445 PMC8947189 35327817

[27] Czajkowski SM, Powell LH, Adler N, , From ideas to efficacy: the ORBIT model for developing behavioral treatments for chronic diseases, Health Psychol. 34 (10) (2015) 971–982. 10.1037/hea0000161 PMC4522392 25642841

[28] Chan AW, Boutron I, Hopewell S, , SPIRIT 2025 statement: updated guideline for protocols of randomised trials, PLoS Med. 22 (4) (2025) e1004589. 10.1371/journal.pmed.1004589 PMC12037212 40294521

[29] Hopewell S, Chan A-W, Collins GS, , CONSORT 2025 statement: updated guideline for reporting randomized trials, JAMA 333 (22) (2025) 1998–2005. 10.1001/jama.2025.4347 40228499

[30] Harris PA, Taylor R, Minor BL, , The REDCap consortium: building an international community of software platform partners, J. Biomed. Inform. 95 (2019) 103208. 10.1016/j.jbi.2019.103208 PMC7254481 31078660

[31] Center for Genetic and Rare Diseases (GARD) Information Center, GARD List of Rare Diseases. https://rarediseases.info.nih.gov/diseases, 2026. Accessed 1/2/2026.

[32] Zenk SN, Simoni JM, Pérez-Stable EJ, Prioritizing research on social determinants of health—“yes, and…_”_, JAMA Intern. Med. 185 (3) (2025) 253–254. 10.1001/jamainternmed.2024.6391 39621326

[33] National Institute on Nursing Research, Social Determinants of Health (SDoH) Survey, National Institues of Health Biomedical Research Informatics Computing System, 2016.

[34] Poverty and child health in the United States, Pediatrics 137(4), 2016. 10.1542/peds.2016-0339 26962238

[35] National Institute on Nursing Research, Palliative Care for Children: Support for the Whole Family When Your Child Is Living With a Serious Illness, NIH Publication #15-NR-80032015, 2026.

[37] Folkman S, Positive psychological states and coping with severe stress, Soc. Sci. Med. 45 (8) (1997) 1207–1221. 10.1016/s0277-9536(97)00040-3 9381234

[38] Leventhal H, Steele DS, Illness Representations and Coping With Health Threats, in: Handbook of Psychology and Health (Volume IV), 2020.

[39] Folkman S, Stress: Appraisal and coping, in: Gellman MD, Turner JR (Eds.), Encyclopedia of Behavioral Medicine, Springer New York, New York, NY, 2013, pp. 1913–1915.

[40] Sonney JT, Insel KC, Reformulating the common sense model of self-regulation: toward parent-child shared regulation, Nurs. Sci. Q. 29 (2) (2016) 154–159. 10.1177/0894318416630091 26980895

[41] Donovan HS, Ward S, A representational approach to patient education, J. Nurs. Scholarsh. 33 (3) (2001) 211–216. 10.1111/j.1547-5069.2001.00211.x 11552546

[42] Aoun SM, Gill FJ, Phillips MB, , The profile and support needs of parents in paediatric palliative care: comparing cancer and non-cancer groups, Palliat. Care Soc. Pract. 14 (2020) 2632352420958000. 10.1177/2632352420958000 PMC7525220 33033802

[43] Aoun SM, Grande G, Howting D, , The impact of the carer support needs assessment tool (CSNAT) in community palliative care using a stepped wedge cluster trial, PloS One 10 (4) (2015) e0123012. 10.1371/journal.pone.0123012 PMC4388632 25849348

[44] Pecanac KE, Repenshek MF, Tennenbaum D, Hammes BJ, Respecting choices® and advance directives in a diverse community, J. Palliat. Med. 17 (3) (2014) 282–287. 10.1089/jpm.2013.0047 24325558

[45] Walter JK, Rosenberg AR, Feudtner C, Tackling taboo topics: how to have effective advanced care planning discussions with adolescents and young adults with cancer, JAMA Pediatr. 167 (5) (2013) 489–490. 10.1001/jamapediatrics.2013.1323 23478910

[46] Cooper B, Kinsella GJ, Picton C, Development and initial validation of a family appraisal of caregiving questionnaire for palliative care, Psychooncology 15 (7) (2006) 613–622. 10.1002/pon.1001 16287207

[47] Saunders R, Moinian D, Stott J, , Measurement invariance of the PHQ-9 and GAD-7 across males and females seeking treatment for common mental health disorders, BMC Psychiatry 23 (1) (2023) 298. 10.1186/s12888-023-04804-x PMC10148535 37118684

[48] Pranckeviciene A, Saudargiene A, Gecaite-Stonciene J, , Validation of the patient health questionnaire-9 and the generalized anxiety disorder-7 in Lithuanian student sample, PloS One 17 (1) (2022) e0263027. 10.1371/journal.pone.0263027 PMC8794093 35085349

[49] Murphy PE, Canada AL, Fitchett G, , An examination of the 3-factor model and structural invariance across racial/ethnic groups for the FACIT-Sp: a report from the American Cancer Society’s study of Cancer survivors-II (SCS-II), Psychooncology 19 (3) (2010) 264–272. 10.1002/pon.1559 19367561

[50] Johnstone B, Yoon DP, Franklin KL, Schopp L, Hinkebein J, Re-conceptualizing the factor structure of the brief multidimensional measure of religiousness/spirituality, J. Relig. Health 48 (2) (2009) 146–163. 10.1007/s10943-008-9179-9 19421866

[51] Health Resources and Services Administration, Rural Health Grants Analyzer. https://data.hrsa.gov/topics/rural-health/rural-health-eligibility. Accessed 1/1/2026.

[52] Kroenke K, Spitzer RL, Williams JB, The PHQ-9: validity of a brief depression severity measure, J. Gen. Intern. Med. 16 (9) (2001) 606–613. 10.1046/j.1525-1497.2001.016009606.x PMC1495268 11556941

[53] Aoun SM, O’Brien MR, Knighting K, Using the carers’ alert thermometer tool to identify needs and support family caregivers of people with motor neurone disease: moving beyond needs assessments, Palliat Care Soc Pract. 18 (2024) 26323524241228306. 10.1177/26323524241228306 PMC10860492 38347888

[54] Ananth P, Melvin P, Berry JG, Wolfe J, Trends in hospital utilization and costs among Pediatric palliative care recipients, J. Palliat. Med. 20 (9) (2017) 946–953. 10.1089/jpm.2016.0496 28453361

[55] Spratling R, Powers E, Development of a data abstraction form: getting what you need from the electronic health record, J. Pediatr. Health Care 31 (1) (2017) 126–130. 10.1016/j.pedhc.2016.07.007 27575272

[56] Lesage FX, Berjot S, Deschamps F, Clinical stress assessment using a visual analogue scale, Occup. Med. (Lond.) 62 (8) (2012) 600–605. 10.1093/occmed/kqs140 22965867

[57] Dallas RH, Kimmel A, Wilkins ML, , Acceptability of family-Centered advanced care planning for adolescents with HIV, Pediatrics 138 (6) (2016). 10.1542/peds.2016-1854 PMC5127070 27940700

[58] Green P, MacLeod CJ, SIMR: an R package for power analysis of generalized linear mixed models by simulation, Methods Ecol. Evol. 7 (4) (2016) 493–498.

[59] Kumle L, Võ ML, Draschkow D, Estimating power in (generalized) linear mixed models: an open introduction and tutorial in R, Behav. Res. Methods 53 (6) (2021) 2528–2543. 10.3758/s13428-021-01546-0 PMC8613146 33954914

[60] Rabe-Hesketh S, Skrondal A, Pickles A, Generalized multilevel structural equation modeling, Psychometrika 69 (2) (2004) 167–190.

[61] Walters SJ, Sample size and power estimation for studies with health related quality of life outcomes: a comparison of four methods using the SF-36, Health Qual. Life Outcomes 2 (2004) 26. 10.1186/1477-7525-2-26 PMC421748 15161494

[62] Bolker BM, Brooks ME, Clark CJ, , Generalized linear mixed models: a practical guide for ecology and evolution, Trends Ecol. Evol. 24 (3) (2009) 127–135. 10.1016/j.tree.2008.10.008 19185386

[63] Groll A, Tutz G, Variable selection for generalized linear mixed models by L1-penalized estimation, Stat. Comput. 24 (2012) 137–154.

[64] Nakagawa S, Schielzeth H, A general and simple method for obtaining R2 from generalized linear mixed-effects models, Methods Ecol. Evol. 4 (2) (2013) 133–142.

[65] Carpenter JR, Goldstein H, Rasbash J, A novel bootstrap procedure for assessing the relationship between class size and achievement, J. Royal Stat. Soc. Series C (Applied Statistics). 52 (2003).

[66] Jaeger TF, Categorical data analysis: away from ANOVAs (transformation or not) and towards logit mixed models, J. Mem. Lang. 59 (4) (2008) 434–446. 10.1016/j.jml.2007.11.007 PMC2613284 19884961

[67] Smeltzer MP, Ray MA, Statistical considerations for outcomes in clinical research: a review of common data types and methodology, Exp. Biol. Med. (Maywood) 247 (9) (2022) 734–742. 10.1177/15353702221085710 PMC9134761 35492022

[68] Bono R, Alarcón R, Blanca MJ, Report quality of generalized linear mixed models in psychology: a systematic review, Front. Psychol. 12 (2021). 10.3389/fpsyg.2021.666182 PMC8100208 33967923

[69] Zhang Z, Multiple imputation with multivariate imputation by chained equation (MICE) package, Ann. Transl. Med. 4 (2) (2016) 30. 10.3978/j.issn.2305-5839.2015.12.63 PMC4731595 26889483

[70] van Buuren S, Groothuis-Oudshoorn K, Mice: multivariate imputation by chained equations in R, J. Stat. Softw. 45 (3) (2011) 1–67.

[71] Hickman SE, Lum HD, Walling AM, Savoy A, Sudore RL, The care planning umbrella: the evolution of advance care planning, J. Am. Geriatr. Soc. 71 (7) (2023) 2350–2356. 10.1111/jgs.18287 PMC10958534 36840690

[72] Sanderson A, Hall AM, Wolfe J, Advance care discussions: Pediatric clinician preparedness and practices, J. Pain Symptom Manage. 51 (3) (2016) 520–528. 10.1016/j.jpainsymman.2015.10.014 26550935

[73] Sisk BA, Bluebond-Langner M, Wiener L, Mack J, Wolfe J, Prognostic disclosures to children: a historical perspective, Pediatrics 138 (3) (2016). 10.1542/peds.2016-1278 PMC5005028 27561728

[74] Yu JA, Bayer ND, Beach SR, Kuo DZ, Houtrow AJ, A National Profile of families and caregivers of children with disabilities and/or medical complexity, Acad. Pediatr. 22 (8) (2022) 1489–1498. 10.1016/j.acap.2022.08.004 36002069

[75] Disorders NOfR, Barriers to Rare Disease Diagnosis, Care, and Treatment in the US: A 30-Year Comparative Analysis, 2020.

[76] Onghena P, Resentful Demoralization, in: Encyclopedia of Statistics in Behavioral Science, 2005.

[77] Feudtner C, Rosenberg AR, Boss RD, , Challenges and priorities for pediatric palliative care research in the U.S. and similar practice settings: report from a Pediatric palliative care research network workshop, J. Pain Symptom Manage. 58 (5) (2019) 909–917.e903. 10.1016/j.jpainsymman.2019.08.011 PMC8499153 31445136

