# Supplementary material for: FACE-Rare: Protocol of a randomized controlled trial of a family-centered advance care planning intervention for families of children with rare diseases
Source: Contemp Clin Trials. Author manuscript; Available in PMC 2026 Nov 14. (PMC13769424; doi:10.1016/j.cct.2026.108430)
Supplement: Supplementary file 1 [file NIHMS2213333-supplement-Supplementary_Material.pdf]

Supplement

Table 1. SPIRIT 2025 checklist of items to address in a randomized trial protocol.

**Section/Topic**	**No.**	**SPIRIT 2025 Item Description**	**Manuscript Location (page)**
**Administrative Information**			
Title	1a	Trial design, population, and intervention	Title Page
Structured Summary	1b	Design, methods, and registration	Abstract
Protocol Version	2	Version date and identifier	N/A
Roles & Responsibilities	3a–d	Names, affiliations, roles of contributors; Trial Sponsor and Role	Title Page
**Open Science**			
Trial Registration	4	Name of registry and ID number	Methods: Trial Design (1)
Protocol and Statistical Analysis Plan	5	Where the analytic plan can be accessed	Methods: Statistical Analysis (12)
Data Sharing	6	Access to deidentified participant data	Supplement
Funding & COI	7a–b	Sources of funding and conflicts	Title Page
Dissemination Policy	8	Plans to communicate trial results	Supplement
**Introduction**			
Background & Rationale	9a	Scientific rationale and summary	Introduction (1)
	9b	Choice of comparator rationale	Methods: Comparison Condition (8)

Objectives	10	Specific objectives/hypotheses	Methods: Aims & Hypotheses (11)
**Methods: Patient and Public Involvement, Trial Design**			
Patient/Public	11	Details of public involvement	Methods: Intervention (6)
Trial Design	12	Type of trial and allocation ratio	Methods: Trial Design (3)
**Methods: Participants, Interventions, and Outcomes**			
Trial Setting	13	Locations where trial is conducted	Methods: Recruitment Procedures (5)
Eligibility Criteria	14a	Eligibility for participants	Methods: Participants (4)
	14b	Eligibility criteria for sites and for individuals	Methods: Facilitator Training (8)
Intervention and Comparator	15a	Intervention and comparator conditions	Methods: Intervention & Comparison Conditions (6)
	15b	Criteria for discontinuing or modifying intervention or comparison for a trial participant	None
	15c	Strategies to improve adherence to intervention/comparator protocols	Methods: Facilitator Training & Fidelity (8)
	15d	Permitted or prohibited concomitant care	Methods: Intervention & Comparison (6)
Outcomes	16	Primary and secondary outcomes	Table 1
Harms	17	Defining and assessing harms	Methods: Safety Monitoring (9)

Participant Timeline	18	Schedule of enrollment/intervention	Figure 1
Sample Size	19	Assumptions and calculation	Methods: Statistical Analysis (12)
Recruitment	20	Strategies for enrollment	Methods: Recruitment Procedures (5)
**Methods: Assignment of Interventions**			
Randomization	21a	Random sequence generation	Methods: Trial Design (3)
	21b	Type of randomization; stratification strategy.	Methods: Trial Design (3)
Concealment	22	Mechanism for concealment	Methods: Trial Design (3-4)
Implementation	23	Who enrolls and assigns	Methods: Trial Design (3-4)
Blinding	24a–c	Who is blinded and unblinding	Methods: Trial Design (3-4)
**Methods: Data Collection, Management, and Analysis**			
Data Collection Methods	25a	Plans for assessment and collection of trial data	Methods: Data Collection (9)
	25b	Plans to promote participant retention and complete follow-up	Methods: Retention Strategies (9)
Data Management	26	Plans for data entry, coding, security, and storage	Methods: Trial Design; Facilitator Training
Statistical Methods	27a	Methods for comparisons for primary and secondary outcomes	Methods: Statistical Analysis (13-14)
	27b	Plans for inclusion in each	Methods: Statistical

		analysis	Analysis (12)
	27c	Missing Data	Methods: Statistical Analysis (14)
	27d	Additional Analyses	Methods: Statistical Analysis (12)
**Monitoring**			
Data Monitoring Committee	28a–b	Data Monitoring Committee details; explanation of interim analyses and stopping guidelines	Methods: Safety Monitoring (9)
Trial Monitoring	29	Frequency and procedures for monitoring trial conduct	Methods: Safety Monitoring (9)
**Ethics**			
Research Ethics Approval	30	Research ethics committee approval	Methods: Trial Design (3)
Protocol Amendments	31	Plans for communicating protocol modifications to relevant parties	N/A
Consent or Assent	32a–b	Consent/assent procedures	Methods: Recruitment Procedures (5)
Confidentiality	33	Collection, Sharing, and Maintenance of Confidentiality of participant personal information	Methods: Trial Design (4)
Ancillary Care	34	Provisions for post-trial care	N/A

Adverse Events (AE), Significant Adverse Events (SAE), and Safety Monitoring.
We have operationalized the definition of an AE and SAE using a satisfaction questionnaire previously developed by the study PI (Lyon) in research of advance care planning with adolescents with HIV and cancer. The definition of an AE includes a participant endorsing, on a satisfaction questionnaire administered after Session 3, that the study intervention was either "too much to handle" or "harmful," and "not useful," "not helpful," "unsatisfactory," and "not worthwhile." The definition of SAE includes emotional distress requiring hospitalization or inpatient behavioral health services felt to very likely related to the study intervention.

Any SAE occurring during the study will be reported and reviewed by the study PI (Lyon). Any SAE will be reviewed to ensure the event was actually caused by the intervention, and not some coincidental issue. These events will be collected and reported in compliance with the local IRB guidelines. We will discontinue the study until a corrective action plan is in place.

To ensure participant safety and well-being, we have established a comprehensive safety plan encompassing rigorous staff training, proactive distress management, and standardized emergency protocols. All research staff undergo intensive training that includes role-playing potential adverse events, and receive clinical supervision from site Co-Investigators. To mitigate fatigue or general emotional distress, study visits can be divided into shorter increments or rescheduled. If a participant becomes overwhelmed, trained staff will provide immediate empathetic support—including involving a trusted hospital staff member if desired—without prematurely encouraging study withdrawal. For acute protocol-related emergencies, such as the endorsement of active psychosis, homicidally, or suicidality during any study phase, research assistants will immediately contact the Principal Investigator and/or site Co-Investigator. The assistant will escort on-site participants to the emergency department or remain with remote participants until a safe hand-off to an appropriate healthcare provider is completed. Non-protocol emergencies, including suspected child abuse or neglect, will be managed according to local institutional protocols and mandated reporting laws. Furthermore, all participants are provided with emergency contact information and have 24/7 access to on-call mental health specialists, multidisciplinary hospital support teams, and referrals for community-based services.

Data Sharing Plan.
The Data Management and Sharing Plan includes the collection of electronic health record data, survey responses, and transcribed interviews from children with rare diseases, their caregivers, and NICU staff, with all data securely stored and de-identified. Coded and aggregated data will be preserved and shared under controlled access through data-sharing agreements that protect participant confidentiality, particularly given the vulnerability of child subjects. Data will be archived institutionally for up to 10 years, documented with detailed metadata and codebooks, and overseen by the Principal Investigator and the Children's National Data Governance Committee. Persons interested in data should contact the PI at mylon@childrensnational.org.

Dissemination Plan.

The dissemination plan focuses on broad, timely, and stakeholder-engaged sharing of study findings. Results will be disseminated through ClinicalTrials.gov, peer-reviewed publications, and presentations at national and international scientific and clinical conferences. To reach non-academic audiences, findings will be shared with family caregivers, advocacy organizations (e.g., NORD), and the public through newsletters, community forums (Science Cafés), and collaboration with community advisory groups, with tailored materials such as digital stories and graphics. Findings will also be translated into actionable messages to inform clinical practice and, when appropriate, policy discussions with advocacy organizations and congressional stakeholders.
